# A Neglected Fracture of Acetabulum Treated With Total Hip Replacement

**DOI:** 10.7759/cureus.61778

**Published:** 2024-06-06

**Authors:** Vishal S Patil, Ishan Shevate, Faiz R Pervez, Dattatray Bhakare

**Affiliations:** 1 Orthopaedics, Dr. D. Y. Patil Medical College, Hospital and Research Centre, Dr. D. Y. Patil Vidyapeeth, Pune (Deemed to Be University), Pune, IND

**Keywords:** open reduction internal fixation, implant, malunion, total hip replacement (thr), neglected acetabulum fracture

## Abstract

Total hip replacement (THR) for osteoarthritis or inflammatory arthritis yields better outcomes than THR for patients with neglected acetabular fractures. The inferior clinical results mostly arise from an unforeseen bone deficit, making the treatment more time-consuming and complex for instances requiring acetabular restoration and bone grafting. There is a lack of research on the clinical results of THR in cases where acetabular fractures have been overlooked.

A 55-year-old male patient presented with a malunited anterior column of the acetabulum, non-union of the posterior column with protrusion, and a significant impaction fracture in the femoral head. He was then treated with open reduction and internal fixation (ORIF) of acetabular columns, along with the use of a reconstruction cage and bone grafting. At the five-year follow-up, the patient had a good outcome.

The keys to success include meticulous preoperative planning using radiography and computed tomography (CT) scans, sufficient exposure to define the fracture pattern, and the availability of a full range of devices and backup implants. If there are any prior implants, they should only be removed if they are infected or in the way of cup implantation. However, if there is a significant amount of bone loss, complex fractures may require extensive repair using revision total hip arthroplasty (THA) implants.

## Introduction

Acute acetabular fractures occur as a consequence of severe trauma, and their treatment is very hard due to the complex three-dimensional structures and anatomical position involved. The range of treatment options varies from a conservative approach to percutaneous fixation to open reduction and internal fixation (ORIF) [1]. However, it still causes significant damage to the hip joint, and research findings showed that surgical treatment yields superior outcomes compared to non-surgical methods [2].

However, there is a scarcity of evidence about the surgical management of neglected acetabular fractures. While it is generally advised not to postpone surgery for more than 11 days, there are instances of overlooked cases in clinical settings, particularly in impoverished nations [3]. The criteria for classifying a fracture as "neglected" are also subject to debate since there are differences in the terminology used in the published literature. However, on the other hand, if a fracture of the acetabulum is beyond a duration of three weeks, then it is classified as ignored. Although it is expected that the outcomes would be worse in these neglected instances owing to extensive callus accumulation, the repercussions of a non-united or malunited acetabular fracture nevertheless have a significant influence on the patient's quality of life [4].

Total hip arthroplasty (THA) is often only performed on patients who have problems with acetabular fractures, namely secondary arthritis and avascular necrosis of the femoral head. Nevertheless, the outcomes of ORIF for complicated and neglected acetabular fractures are reliably worse due to the excessive production of callus and the repercussions of acetabulum fragments that have not properly healed or have healed in an abnormal manner. These consequences include early post-traumatic arthritis, which has a detrimental effect on the patient's quality of life [4].

Treatment for a neglected acetabular fracture differs primarily from that for an acute fracture because of the possibility of malunion of the fracture components. Accurate reduction is essential to obtain a positive functional outcome. However, in instances where fracture fragments get fused in an abnormal position and are ignored for long, it becomes very challenging, or sometimes impossible, to realign them. The potential discoveries include detachable callus, organized non-unions, and malunions [4]. Furthermore, a delay between the injury and the operation decreases the likelihood of attaining a successful realignment, resulting in an unfavorable clinical outcome.

## Case presentation

A 55-year-old male worker presented to the emergency department with left hip discomfort and edema for six hours. After proper investigations, like an X-ray and computed tomography (CT) scan of the hip, a displaced comminuted anterior column fracture with a posteriorly displaced acetabulum fracture was confirmed. The patient was taken up for surgery, but in the operating room, the patient suddenly developed cardiac arrhythmias, and owing to his history of uncontrolled diabetes mellitus with a random plasma glucose level of 491 mg/dL, the anesthesiologist deemed the patient unsuitable for surgery. Due to the inability to undergo surgery, the patient was treated with traction for six weeks and then mobilized with a walker.

**Figure 1:**
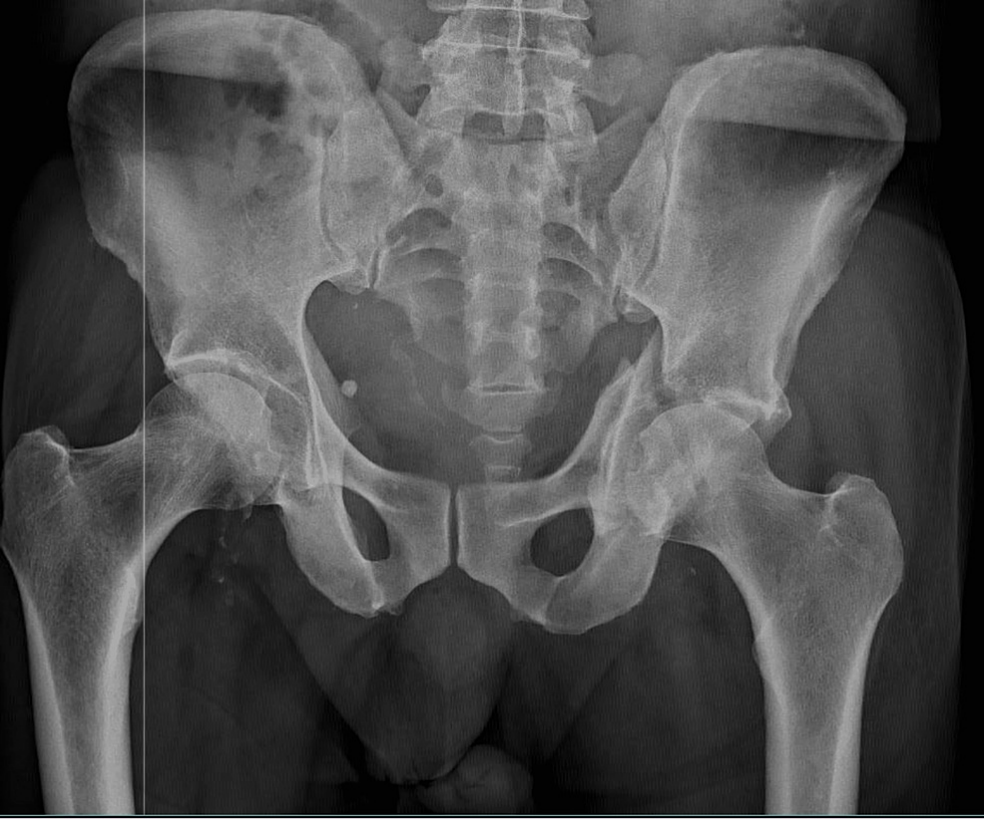
Anterior-posterior X-ray of the pelvis showing non-union of a left acetabular fracture A radiograph of the pelvis of both hips showed that the anterior column and wall had healed together, whereas the posterior column had not healed and showed medial migration and distortion of the femoral head due to an impacted fracture

**Figure 2:**
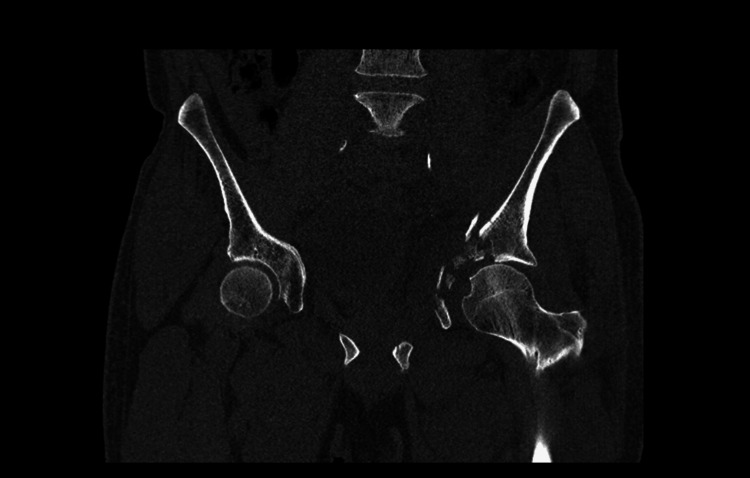
Coronal pelvic CT scan showing non-union of a left acetabular fracture A CT scan of the pelvis showed that the anterior column and wall had healed together, whereas the posterior column had not healed and showed medial migration and a distortion of the femoral head due to an impacted fracture

After one year, the patient reported persistent pain in the left hip joint, particularly while walking. A follow-up radiograph of the pelvis of both hips showed that the anterior column and wall had healed together, whereas the posterior column had not healed and showed medial migration and distortion of the femoral head due to an impacted fracture (Figures 1-2).

At this stage, his medical conditions improved, and he was posted for surgery with high-risk consent. The hip was exposed through the Kocher-Langenbeck (KL) approach. The hip was dislocated, the femoral neck cut was taken, and circumferential capsular release was done. The union of the anterior column was confirmed, whereas the posterior column was in non-union as well as it was medially displaced.

The sciatic nerve and sciatic notch were dissected, and the quadrilateral plate was mobilized. The fibrous tissue in the fracture site was removed, and the fractured posterior column was reduced to reconstruct the acetabulum. The posterior column was stabilized using ORIF with two reconstruction plates. Then the femoral head was mobilized and positioned on the floor of the acetabulum.

A Burch-Schneider reinforcing cage was implanted. A total hip replacement (THR) procedure was conducted using a cemented cup, and an uncemented stem was inserted. The stability of the hip was confirmed intraoperatively. Following the surgery, the patient did not have any neurovascular deficit and was mobilized non-weight bearing with a walker for six weeks. Deep venous thrombosis (DVT) prophylaxis and indomethacin for the prevention of heterotrophic ossification were given. The patient achieved full weight-bearing ambulation within 10 weeks after the operation.

**Figure 3:**
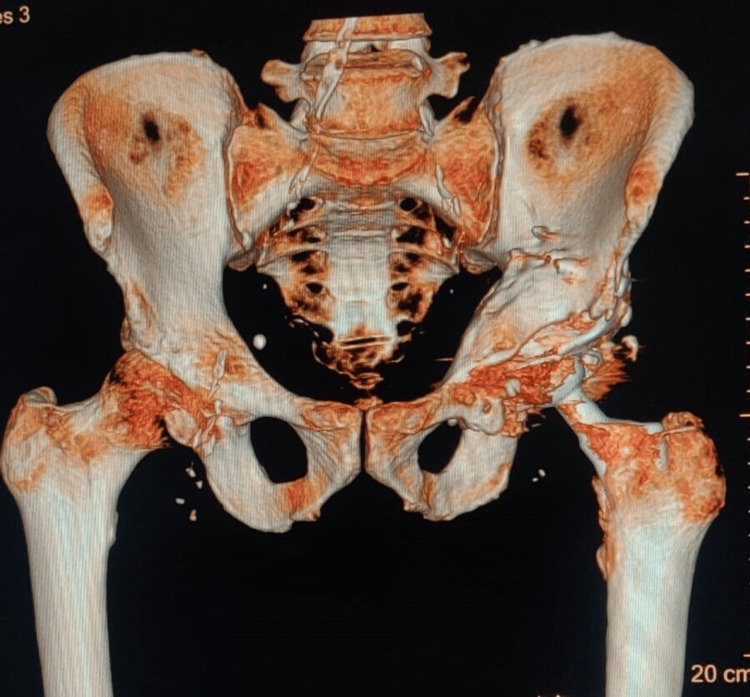
CT scan of the hip during the 12-month follow-up visit showing the union of both columns

The patient was on injection teriparatide for three months. At the 12-month follow-up, a CT scan of the hip confirmed the union of both the anterior and posterior columns (Figure 3).

**Figure 4:**
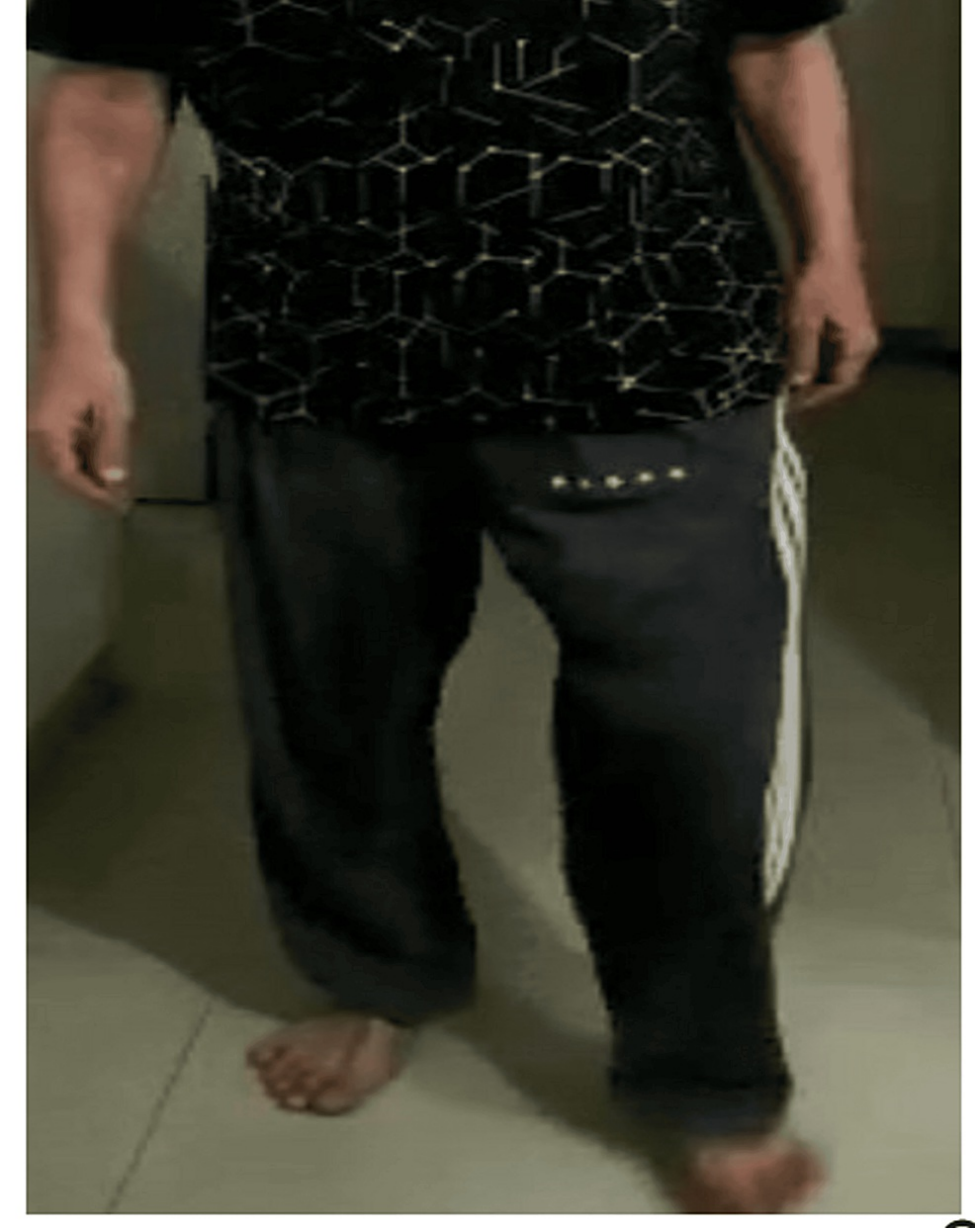
Patient standing during his follow-up after five years

**Figure 5:**
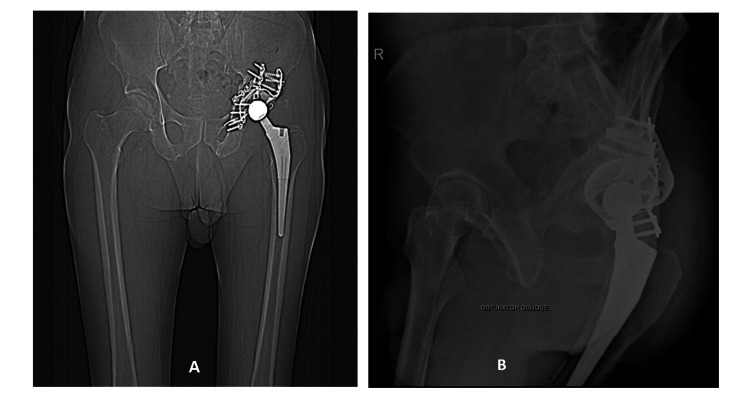
Five-year follow-up radiological imaging shows the implant in situ Panel A: Coronal view of CT scan showing the implant Panel B: Five-year follow-up X-ray of the pelvis with the implant

The last follow-up of the patient was done five years post-surgery, with clinical and radiological examinations (Figures 4-5). The patient experienced a comfortable recovery and was back to his daily work routine and activities. The patient has free and full hip movement with some abductor lurch.

## Discussion

Acetabular fractures are a primary contributor to the development of secondary osteoarthritis, which often necessitates the need for a complete hip replacement. Typically, these injuries are prevalent among young individuals. Ideally, THR should be straightforward; nonetheless, there may be complications that might undermine the effectiveness of the therapy and its outcome [5-7].

When planning hip replacement in acetabular fractures, it is crucial to take into account retained implants, bone defects, nonunion, innominate bone deformity, reduced musculature, heterotopic ossification (HO), and infection. Erythrocyte sedimentation rate (ESR) and C-reactive protein (CRP) must be done to rule out subclinical infection preoperatively.

Acetabular fractures are most commonly fixed by the KL approach (for posterior wall and column fractures) and the combination of the ilioinguinal approach with the Stoppa approach (for anterior column and quadrilateral plate fractures).

During THR in neglected acetabulum fractures, we prefer the posterior approach as it is extensive, and complex reconstruction can be done. Sciatic nerve neurolysis might be required to expose the acetabulum. Previous anterior approaches should not hamper implant positioning in such cases. Pre-operative CT scans must be performed to assess the bone loss and malunion of the acetabulum. This also gives clarity regarding the method to use for the reconstruction of the acetabulum.

Various procedures have been recommended for the reconstruction of acetabular defects. The options available include autograft, allograft, oblong acetabular cups, and trabecular metal augments, with or without reinforcing cages [8]. Bone grafting is an appropriate method for addressing acetabular deformities. Structural grafts may be advantageous in cases when further procedures may be necessary, since they help to restore the center of rotation and bone stock [8]. The inclusion of vascularization and osteointegration provides further advantages by reducing the likelihood of loosening and dislodgement.

Bone grafting is the preferred method for protrusion deformity, so bone graft and synthetic graft substitutes must be ready. Acetabulum augments must be kept for the reconstruction of non-contained defects. As the tantalum acetabulum augments are not available, anti protrusion cage can be used as demonstrated in this case. In cases of pelvic discontinuity which is chronic in cases of revision THR, a cup cage construct is preferred. But in the case of acetabular fractures, this discontinuity can be fixed, and bone can be grafted similarly to any other nonunion.

After fixing the acetabulum, if there is no significant bone loss in the wall, a multi-hole cup with screws in different columns can be used for the reconstruction of the acetabulum.

In most cases, a neglected acetabular fracture requires reconstructive surgery when there is a lack of alignment between the acetabular cup and the femoral head [4]. Acetabular fractures require exact bone alignment, just like any other joint fractures, and it has been demonstrated that establishing better alignment leads to more favorable clinical outcomes [3]. The necessity for a THR increases with insufficient acetabular reduction, according to research by Bastian et al. [9]. It makes sense that the goal in acute acetabular fracture cases should be to achieve anatomical alignment in order to delay degenerative changes that could otherwise necessitate an accelerated hip replacement. Research on acetabular fractures in 424 patients revealed that, despite being performed by skilled surgeons, 9-12% of these fractures were inadequately reduced [3]. The factors that contribute to inadequate reduction include the presence of associated fractures, advanced age, and a delay in undergoing surgery. Acetabular fractures that have been neglected, non-united, or malunited are making it challenging to visually identify the fracture pattern and reduction during surgery [4].

Proper positioning of an acetabular cup in such a deformed acetabulum requires expertise. Robotic hip replacement surgery can aid in the accurate positioning of the pelvis in such a deformed pelvis.

A study of 128 individuals with fractures in the posterior wall of the acetabulum revealed that over 50% of those aged over 50 and had marginal impaction and wall comminution required hip arthroplasty at an early stage. The study findings suggested prompt THA for individuals in this age range. Many studies have shown various factors that might predict failure after reduction and internal fixation in senior individuals. Hip dislocation, comminuted fractures of the posterior wall, impaction of the superomedial dome or the posterior wall, and injury to the femur head are among these causes [10]. The surgeon has to make sure that the acetabular fragments are sufficiently stable for the acetabular component to fit properly before attempting early arthroplasty [11].

Although acetabular reinforcement cages and ceramic-on-ceramic bearing surfaces may offer better long-term durability for neglected acetabular fractures, we used standard acetabular cups and ceramic-on-polyethylene bearing surfaces in all of our arthroplasty cases. Dual mobility liners have been shown to reduce the risk of dislocations.

Acetabular fractures may lead to post-traumatic nerve palsies in around 16.4%, and according to research during an open surgical approach, the incidence of damage to the sciatic nerve and the femoral nerve is estimated to be around 5-6% [12] and 0.3% [13], respectively. These challenges emphasize the need for proper preoperative strategies and precise scheduling of the surgery [14,15].

The study by Chémaly et al. [16] indicated that the risk of developing severe HO was significantly increased in early (acute) THA; the other studies did not report such a difference.

In our case, proper planning, control over medical conditions, reconstruction of the acetabulum, and bone grafting have given good support for the implant to survive for a longer duration.

## Conclusions

Our case report findings indicate that THR might be a viable and successful treatment option for untreated acetabular fractures. Undervalued concomitant fracture types provide more challenges in terms of repair and exhibit worse surgical results as compared to basic forms. Favorable results are anticipated when a strong bone foundation is established via the use of several surgical methods. Our exploration of neglected acetabular fractures emphasizes the intricate balance between surgical precision, technological innovation, and patient-centered care. Through meticulous preoperative evaluation and tailored reconstruction strategies, we aim to address anatomical complexities and optimize long-term outcomes. While challenges such as nerve injuries and HO should be overpowered by vigilant management, advancements in techniques, such as robotic assistance, can also play a significant role. By embracing interdisciplinary collaboration and adhering to evidence-based practices, we strive to achieve durable results and ultimately improve the quality of life for patients undergoing THR in the context of neglected acetabular fractures.
